# Effects of Neural Mobilization on Sensory Dysfunction and Peripheral Nerve Degeneration in Rats With Painful Diabetic Neuropathy

**DOI:** 10.1093/ptj/pzac104

**Published:** 2022-08-01

**Authors:** Guan-Cheng Zhu, Yu-Wen Chen, Kun-Ling Tsai, Jhi-Joung Wang, Ching-Hsia Hung, Annina B. Schmid

**Affiliations:** 1 Department of Physical Therapy, National Cheng Kung University, Tainan, Taiwan (R.O.C.); 2 Department of Physical Therapy, China Medical University, Taichung, Taiwan (R.O.C.); 3 Department of Medical Research, Chi-Mei Medical Center, Tainan, Taiwan (R.O.C.); 4 Nuffield Department of Clinical Neuroscience, University of Oxford, Level 6, West Wing, John Radcliffe Hospital, Oxford OX3 9DU, U.K.

**Keywords:** Diabetic Neuropathies, Nerve Degeneration, Pain Management

## Abstract

**Objective:**

This study aims to evaluate the effectiveness of neural mobilization (NM) in the management of sensory dysfunction and nerve degeneration related to experimental painful diabetic neuropathy (PDN).

**Methods:**

This is a pre-clinical animal study performed in the streptozocin (STZ)-induced diabetic rat model. 3 groups were included; a treatment group of rats with PDN receiving NM under anesthesia (PDN-NM, n=10), a sham treatment group of rats with PDN that only received anesthesia (PDN-Sham, n=9) and a vehicle control group with nondiabetic animals (Vehicle, n=10). Rats in the PDN-NM and PDN-Sham groups received 1 treatment sessions on day-10, 12 and 14 after STZ injection, with **a** 48-hour rest period between sessions. Behavioral tests were performed using von Frey and Plantar tests. Evaluation for peripheral nerve degeneration was performed through measuring protein gene product 9.5-positive (PGP9.5+) intra-epidermal nerve fiber density (IENFD) in hind-paw skin biopsies. All measurements were performed by a blinded investigator.

**Results:**

The behavioral tests showed that a single NM session could reduce hyperalgesia which was maintained for 48 hours. The second treatment session further improved this treatment effect, and the third session maintained it. These results suggest that it requires multiple treatment sessions to produce and maintain hypoalgesic effects. Skin biopsy analysis showed that the PGP9.5+ IENFD was higher on the experimental side of the PDN-NM group compared to the PDN-Sham group, suggesting NM may mitigate the degeneration of peripheral nerves.

**Conclusions:**

This study demonstrated that NM may be an effective method to manage experimentally induced PDN, potentially through mitigation of nerve degeneration.

Further studies are needed to develop standardized protocols for clinical use.

**Impact:**

These findings provide neurophysiological evidence for the use of NM in PDN and can form the basis for the development of physical therapy-based programs in clinics.

## Introduction

Diabetic neuropathy (DN) is 1 of the most common diabetes-related complications. Approximately 25%–50% of patients with diabetes seek medical attention for the management of DN.^1, 2^ The clinical manifestation of DN includes loss of sensory function that manifests through symptoms like numbness or decreased sensory perception^3, 4^ and gain of sensory function, which associated with neuropathic pain-like symptoms including spontaneous pain and mechanical/thermal hypersensitivity.^5–13^ The gain of function is colloquially known as painful diabetic neuropathy (PDN), and is the most common cause of chronic pain in diabetes.^1, 2^ PDN severely affects quality of life and its treatment is resource intense.^1, 2, 14^ Thus, a cost-effective management strategy for PDN is critical for improving the quality of care for patients and improving the efficient use of medical resources.^15, 16^

The management of PDN remains challenging. Current first-line treatment for PDN includes anticonvulsive and anti-depressant drugs.^10^ However, these medications only provide 30%–50% pain relief and are often accompanied by unwanted side effects.^30, 17–19^ One reason why treatments for PDN remain mostly ineffective is because the physiological mechanisms of PDN remain unclear. Recent studies suggest that degeneration and regeneration of peripheral nerves is an important pathway in the development of PDN.^7, 10, 20–22^ Degeneration of peripheral nerves is common in diabetes as apparent by reduced nerve conduction velocities and decreased intra-epidermal nerve fiber densities (IENFD) in skin biopsies.^10, 20, 23^ It is also common that degeneration and regeneration of peripheral nerves co-exist.^24–26^ Current consensus is that the loss of sensory function is likely the result of nerve fiber loss^10, 20^, while the gain of sensory function may be influenced by both axon degeneration and the concomitant regeneration of unmyelinated fibers.^22, 24, 27^ Thus, treatments that mitigate progressive peripheral nerve degeneration might be promising for the effective management of PDN.^10^

One limitation of current pharmacological treatments for PDN is that they target nociception, not the mitigation of nerve degeneration. Recent preclinical and clinical studies using nonpharmacological interventions like exercise show promising results in mitigating sensory dysfunction related to PDN^28–31^ while also suggesting a potential regenerative ability in focal nerve injury.^32–35^ One example of such exercise is neural mobilization (NM). NM utilizes limb movements designed to move peripheral nerves relative to their surrounding structures.^36^ Preclinical studies suggest that NM mitigates neuropathic pain through modulating neuroinflammation, the endogenous opioid system and neurotrophic factors.^28, 37, 38^ There is also growing evidence that NM may promote nerve regeneration after focal nerve injury.^32, 33^ To date, the neurophysiological effect of NM in systemic neuropathies such as PDN remain elusive. Our previous study found that 3-weeks of daily NM treatment alleviated mechanical allodynia in rats with PDN.^28^ However, the temporal relationship between NM treatments and hypoalgesia as well as a potential pro-regenerative effect of NM on peripheral neurons in PDN remains to be examined.

This study has 2 objectives. First, we aim to analyze the temporal pattern of behavioral responses associated with NM interventions in PDN to investigate the effect duration of single NM session and the potentiation effect of multiple NM sessions. This will help to develop more efficient treatment programs for clinical use. The second objective is to investigate whether NM can mitigate the degeneration of peripheral nerves related to PDN. This will provide further evidence for the neurophysiological benefits of NM in managing PDN.

## Methods

### Ethics Approval

All experimental procedures followed the Guideline for the Care and Use of Laboratory Animals, Taiwan (R.O.C.) and were approved by the Institutional Animal Care and Use Committee (IACUC) of National Cheng Kung University. IACUC approval number 108142.

### Animal model

The streptozocin (STZ)-induced PDN rat model was used for this study.^28, 29^ Mature (8-weeks old) male Sprague-Dawley (SD) rats weighing 280–300g from BioLASCO were divided into 3 groups; a vehicle control group (Vehicle), a PDN with NM group (PDN-NM) and a sham treatment group (PDN-Sham group). Only male rats were used in this study to minimize the influence of estrous cycle on pain sensitivity in female rats.^39^

Animals were housed in clear cages with shredded wood chips, the cages were changed daily and the housing facility maintained constant room temperature at 25±2°C and humidity (50%) with unrestricted food/water access unless otherwise specified. Two to 3 rats were housed together and environment enrichment were provided to prevent loneliness and stress. Rats were fasted overnight before STZ injection to induce diabetes.^40^ Before injection, rats were randomly assigned using Microsoft Excel to receive STZ or saline injection. The rats were anesthetized with intraperitoneal pentobarbital injection (35mg/kg) and STZ/saline was injected into the femoral vein (65mg/kg). A blood glucose test was performed 3 days after STZ injection to confirm the development of diabetes; rats with fasting (8 hours) blood glucose level over 300mg/dl were considered to have developed diabetes.

### General procedure and experimental groups

**Figure 1:**
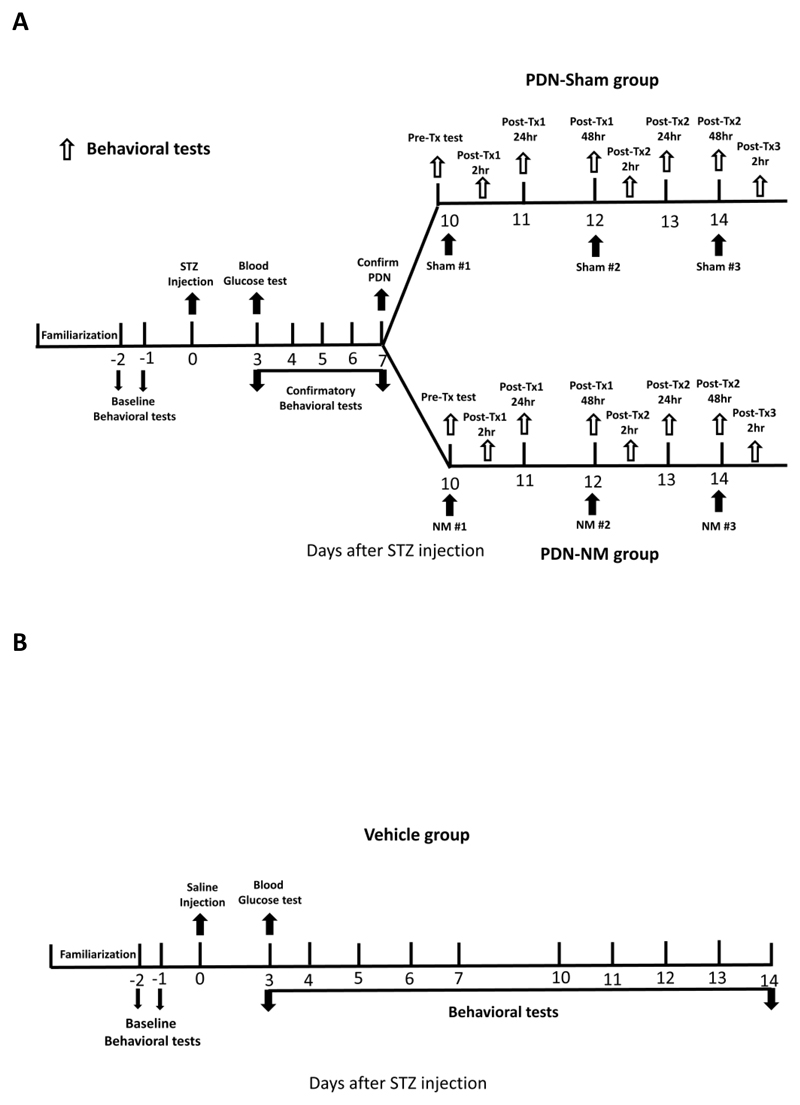
General procedure of this study. (A) Procedure for rats in the PDN-Sham and PDN-NM groups. After baseline behavioral tests and STZ injection, the rats received 5 days of confirmatory behavioral tests to monitor the development of PDN. The rats with decreased mechanical or thermal response threshold at day-7 post-injection were considered to have developed PDN and were randomly assigned to PDN-Sham or PDN-NM groups. The rats in PDN-Sham and PDN-NM groups received 3 treatment sessions on days 10, 12 and 14 post-STZ injection. Behavioral tests to monitor the treatment effect were performed before each treatment session and at 2, 24, and 48 hours after the first and second treatment session and 2 hours after the third treatment session. (B) Procedure for rats in the Vehicle group. After baseline behavioral tests and saline injection, the rats in the Vehicle group received daily behavioral tests.

The general procedure of this study is summarized in Figure 1. Rats underwent environment familiarization for 3 days before baseline testing. After familiarization, 2 baseline behavioral tests at 2/1 day before injection were performed and the data were averaged. After STZ/saline injection, confirmatory behavioral tests were performed daily from day-3 to day-7 after injection to confirm the development of PDN. Diabetic rats with decreased mechanical response thresholds at day-7 compared to baseline were considered to have developed PDN.^29^ The rats with PDN were randomly assigned using Microsoft Excel to PDN-NM or PDN-Sham groups whereas the rats that did not develop PDN were excluded from the study. Throughout the study, all rats received behavioral tests to monitor their sensory profile. The rats in the PDN-NM and PDN-Sham groups received NM under isoflurane anesthesia (PDN-NM) or sham treatment consisting of only isoflurane anesthesia (PDN-Sham). A total of 3 treatment sessions were given on day 10, 12, and 14 after STZ injection. Behavioral tests to monitor the treatment effect of sham treatment and NM were performed before each session and at 2, 24, and 48 hours after the first and second treatment session and 2 hours after the third treatment session (Fig. 1A).

This design allowed us to evaluate the effect duration of a single NM session and the potentiation effect of multiple NM sessions. Rats in the Vehicle group received daily behavioral tests to monitor their sensory profile (Fig. 1B).

### Interventions

The NM treatment was performed on the right hind-leg following the protocol described in our previous study.^28^ For clarity, the right/left sides are described as “experimental side” and “contralateral side”. Rats were anesthetized with 3% isoflurane gas to minimize distress. During treatment, the rats were positioned side-lying with hip fixed at 70–80 degrees flexion and knee at full extension to put the sciatic nerve and its branches in a lengthened position. To produce further tension, the rats’ ankle was oscillated into dorsiflexion/plantar flexion. The range of motion was set at 20–25 degrees from neutral in both directions and was performed at a pace of once every 2 seconds for 2 minutes, followed by a 30-second break. This was repeated five times with the total time for 1 session being approximately 12.5 minutes. This NM technique was modified from the method described by Santos et al.^37, 38^ We did not perform the cervical flexion maneuver as we considered this to induce too much tension which may not be tolerated in a clinical setting. A single NM/Sham treatment session was given on 3 separate days (day 10, 12, and 14 after STZ injection). The rats in the PDN-Sham group received isoflurane anesthesia for the same duration and timepoints but did not receive any NM treatment.

### Behavioral tests

Mechanical and thermal behavioral tests were performed on bilateral hind-paws by an investigator blinded to group allocation. Mechanical response thresholds were measured with von Frey filaments using a method based on the simplified up-down method (SUDO)^38, 41^ described in our previous studies.^28, 29, 42^ For mechanical response thresholds, rats were put on a wire mesh table with a clear polymer cage. Monofilaments (Touch Test) weighing 0.6–26g were used to stimulate the hind paw at the center of the walking pads, an area innervated by the tibial nerve.^43, 44^ Each rat was tested twice and the response thresholds (grams at paw withdrawal) were averaged.^29^ The averaged mechanical response threshold was converted into a log(10) scale using this equation: Log_10_(mechanical response threshold in 1/10mg)

Thermal response thresholds were measured using the Hargreaves’ method.^29, 45^ Animals were placed in a clear polymer chamber with breathing holes on a glass plate that was maintained at 25±1°C. A heat lamp was used to apply heat on the hind-paw and the withdrawal latency was measured. The withdrawal responses included foot lifting, licking and squeaking. To prevent heat damage to the paws, an automatic cut-off was set at 20 seconds. The test was repeated 3 times and the response latency (seconds to paw withdrawal) was averaged.

### Nerve fiber integrity on skin biopsies

After the final behavioral test, rats were sacrificed to collect tissue for immuno-histochemical (IHC) analysis. Rats were deep-anesthetized with urethane (1.4g/kg) and sacrificed by heart perfusion with saline followed by 30 minutes of 4% paraformaldehyde solution for fixation. Glabrous plantar skin from the center of both hind paws was collected and preserved in 4% paraformaldehyde solution for 24 hours before transferring into 30% sucrose solution for 3 days and embedding in OCT and freezing at -80°C.

Fourteen micrometer (14μm) sections were cut on a cryostat (Thermo Fisher Scientific, Waltham, MA, U.S.A.) and mounted on gelatinized slides. Sections were dried at room temperature for 1 hour before being incubated in blocking solution (5% normal donkey serum, 1% bovine serum albumin, 1% dimethyl sulfoxide, 0.5% milk powder, 0.3% Triton X-100, 0.1% sodium azide in PBS) at room temperature for 2 hours. Sections were then incubated with primary antibody of protein gene marker 9.5 (PGP9.5, a pan axonal marker) overnight at 4°C. After that, the sections were washed with washing buffer (0.05% Triton X-100 in PBS) and horse anti-mouse biotinylated antibody was added and incubated at room temperature for 2 hours. After another wash, secondary streptavidin fluorescent antibody was added and incubated at 4°C for 2 hours. The sections were then washed again and mounted with Vectashield mounting medium (Vector laboratories, Burlingame, CA, U.S.A.). The Table summarizes the information for the antibodies.

The stained sections were viewed and counted under a fluorescence microscope (Olympus BX51, Center Valley, PA). PGP9.5 positive (PGP9.5+) intra-epidermal nerve fibers that crossed the dermal–epidermal junction were counted, adhering to published principles.^46^ Photographs of sections were then taken using an Olympus DB80 camera (Olympus, Center Valley, PA) and the length of the epidermis was measured by Olympus Cell Sense software (Olympus, Center Valley, PA). Intra-epidermal nerve fiber density (IENFD) was expressed as fibers/mm epidermis. Data from 3 sections were averaged for each animal. Evaluation of IENFD was performed with the investigator blinded to group allocation.

### Statistical analysis

All statistical analyses were performed with SPSS 17.0. Normality of data was checked by inspection of histograms. The daily behavioral data were analyzed with separate 2 way (time, group) mixed model analyses of variance (ANOVA) for the experimental and contralateral sides. Significant omnibus tests were followed by post-hoc Fisher tests to compare the behavioral data between different timepoints within each group. In addition, post-hoc Fisher tests were used to compare the difference in behavioral data between groups at each timepoint.

The fine-grained behavior data in relation to intervention time points (time-course test) for PDN-Sham and PDN-NM groups were separately analyzed with the same method to investigate group differences over time, pairwise differences of behavioral data between PDN-NM and PDN-Sham groups at each timepoint and the difference between timepoints within each group.

The IENFD data were analyzed using 1-way ANOVA followed by post-hoc Fisher test to evaluate group differences.

Mauchly’s test was used to assess the assumption of sphericity. If the assumption of sphericity was not met, we report the main effect with Huynh-Feldt (ε > 0.75) or Greenhouse-Geisser correction (ε < 0.75). Significance was set at *P* < .05.

### Role of the funding source

This study was funded by the Ministry of Science and Technology (MOST), Taiwan (R.O.C.), 108-2314-B-006 -070 -MY3. Guan-Cheng Zhu is supported by the MOST funding for Recruitment of Visiting Science and Technology Personnel, 110-2811-B-006-509-/110-2811-B-006-523-. Annina B. Schmid is supported by a Wellcome Trust Clinical Research Career Development Fellowship (222101/Z/20/Z). The funders played no role in the design, conduct, or reporting of this study.

## Results

### Number of animals

In total 36 rats were used in this study. Eleven rats received saline injection. Of these, 1 showed mechanical allodynia for unknown reasons and was excluded from the study, the other 10 rats were included in the Vehicle group. Twenty-five rats received STZ injection. Of these, 3 did not develop diabetes and 2 died during anesthesia and 1 died immediately after STZ injection. The remaining 19 rats developed PDN and were randomly allocated to PDN-Sham (n=9) and PDN-NM group (n=10).

### PDN rats develop reduced mechanical, but not thermal response thresholds

The daily behavioral data are summarized in Supplementary Table 1.

**Figure 2:**
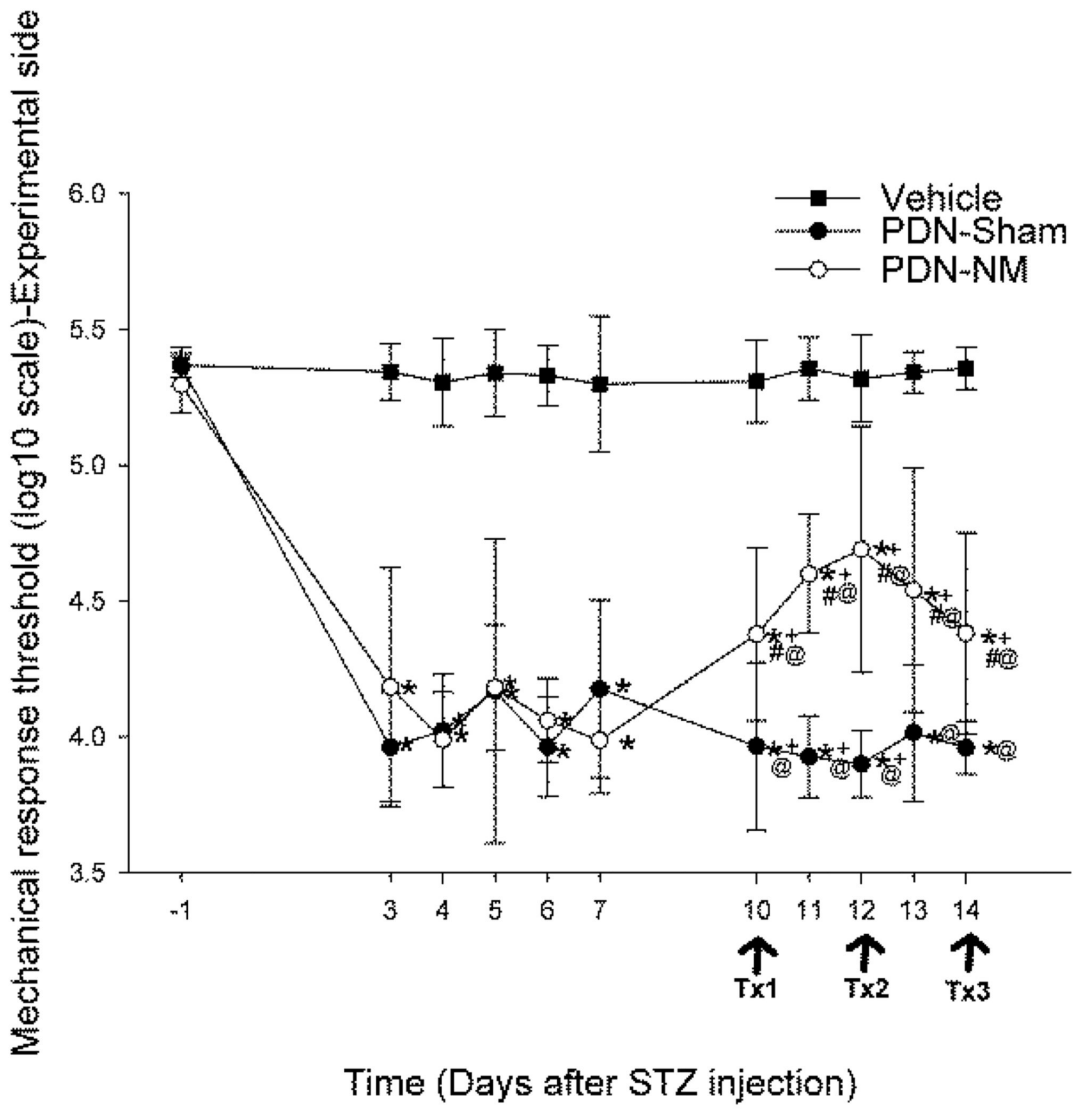

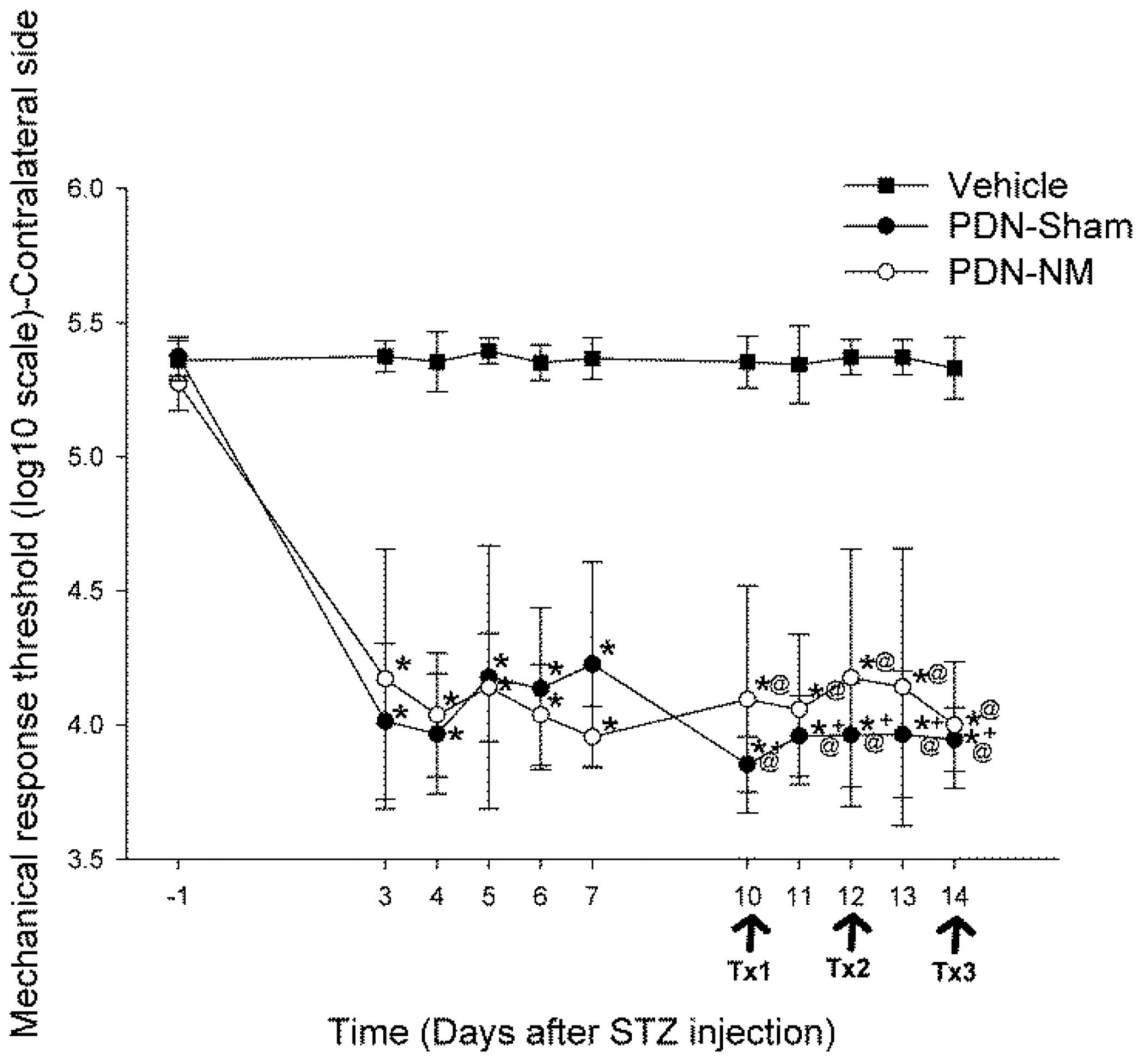

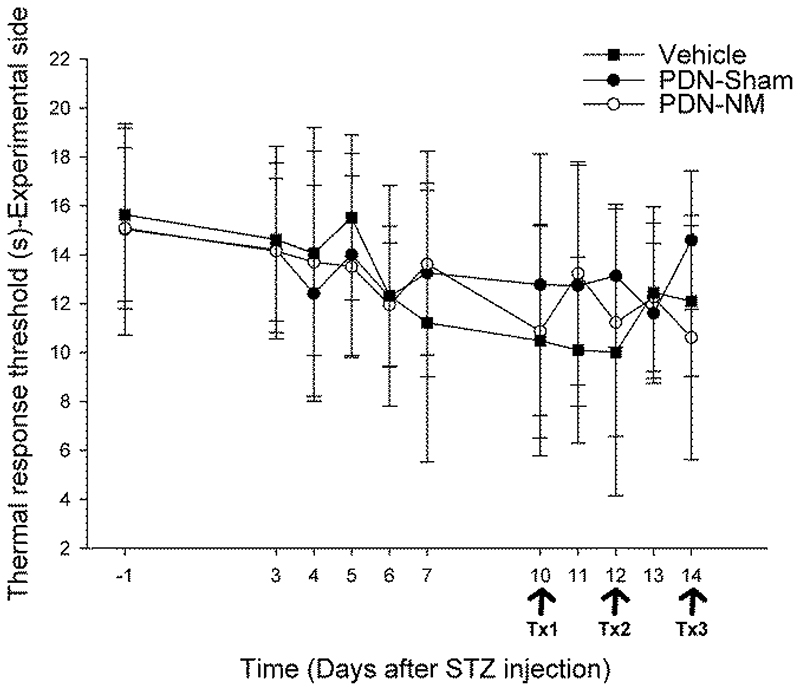

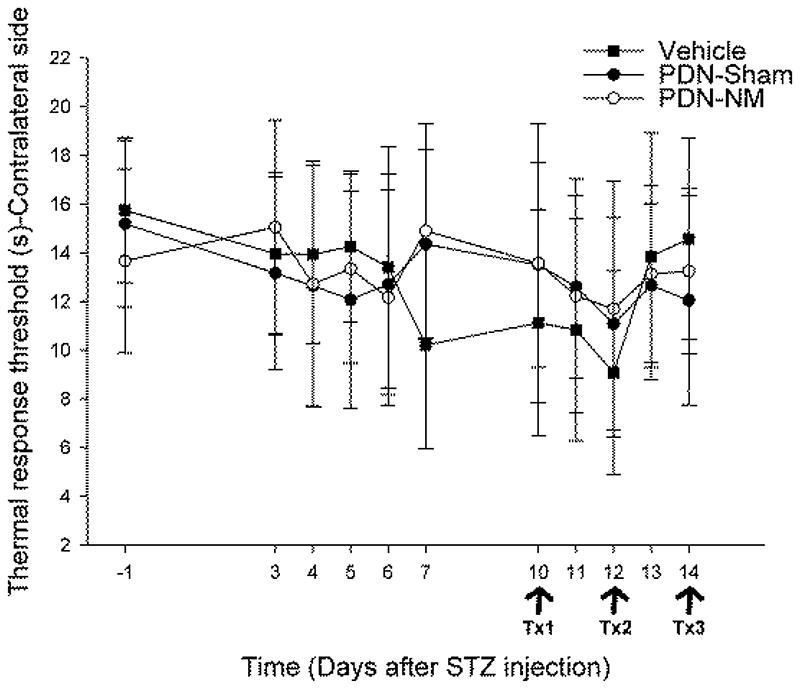
Mechanical von Frey (A, B) and thermal plantar test (C, D) data from the experimental (A,C) and contralateral (B,D) paws. The x-axis represents time, y-axis represents the mechanical response threshold (log(10) scale) and thermal response latency (s). Tx1, Tx2 and Tx3 represent the first, second and third treatment session. Data are presented as mean ± standard deviations with 9 rats in PDN-Sham, 10 in PDN-NM, and 10 in Vehicle group. The mechanical response threshold of PDN-Sham and PDN-NM group showed significant reduction after STZ injection (A, B). The rats in the PDN-NM group showed improvement in the mechanical response threshold on the experimental but not contralateral side starting on day-10 after the first treatment session (A). As for the thermal response threshold, no significant differences were apparent between groups. *P < .05 compared to baseline data, +*P* < .05 compared to pretreatment data from day-7 after STZ injection, #*P* < .05 compared to PDN-Sham group at the same timepoint, @*P* < .05 compared to Vehicle group at the same timepoint.

The result of the 2-way mixed ANOVA for mechanical response thresholds showed that there were significant time-by-group interaction effects on both experimental and contralateral sides (*P* < .001). Post-hoc analyses comparing within-group changes at different timepoints revealed that the mechanical response threshold in the PDN-Sham and PDN-NM groups was significantly decreased bilaterally from day 3 after STZ injection and remained lower until the end of the study compared to baseline (*P* < .001), while the mechanical response threshold of the Vehicle group remained unchanged (Fig. 2A, 2B). For thermal hyperalgesia, the 2-way mixed ANOVA showed no significant time-by-group interaction on both experimental and contralateral sides (*P* = .368/0.142, Fig. 2C and 2D). These results indicate that the rats with PDN developed significant mechanical, but not thermal hypersensitivity.

### Neural mobilization improves mechanical but not thermal response thresholds in PDN rats

Fisher post-hoc tests were used to identify differences between pretreatment (day 7) and post-treatment timepoints (days 10–14) within PDN-Sham and PDN-NM groups. The result showed that the mechanical response threshold of the PDN-NM group was significantly increased on the experimental side on days 10–14 compared to pretreatment baselines (*P* < .001 for days 10–13, *P* = .001 for day 14, Fig. 2A). In the PDN-Sham group, the response thresholds were significantly reduced on days 10–12 compared to day 7 (*P* = .016, 0.002, and 0.026 respectively, Fig 2A). On the contralateral side, the mechanical response thresholds of the PDN-NM group remained unchanged compared to the pretreatment timepoint, while thresholds in the PDN-Sham group were reduced at all post-treatment timepoints (*P* = .001, 0.006, 0.035, 0.040, and 0.002 for days 10-14, Fig. 2B). This indicates that NM selectively increased the mechanical response threshold on the experimental side of the PDN-NM group while the PDN-Sham group continued to deteriorate over time.

We then compared the behavioral data between 3 groups at pre (day 7) and post-treatment timepoints (day 10-14) with post-hoc Fisher test. The results revealed that the mechanical response thresholds on the experimental side of the PDN-NM group was significantly higher compared to the PDN-Sham group on days 10-14 (*P* = .002 for Day 10, *P* < .001 for Day 11-12 and 14, *P* = .001 for Day 13), but did not reach normal levels seen in the Vehicle group (*P* < .001 at all post-treatment timepoints, Fig. 2A).

On the contralateral side, both PDN-NM and PDN-Sham groups had lower mechanical response thresholds than the Vehicle group (*P* < .001) without differences in the PDN groups at any post-treatment timepoint. This further confirms that NM can effectively alleviate mechanical hypersensitivity in rats with PDN on the experimental side. For the thermal response thresholds, there was no significant time-by-group interaction effect (*P* = .368/0.142 for the experimental/contralateral sides, Fig. 2C and 2D). This suggests that neither the STZ injection nor the NM or sham intervention modified the thermal pain sensitivity in rats with PDN.

### Effect duration of a single |NM session and additive effect of multiple sessions

**Figure 3:**
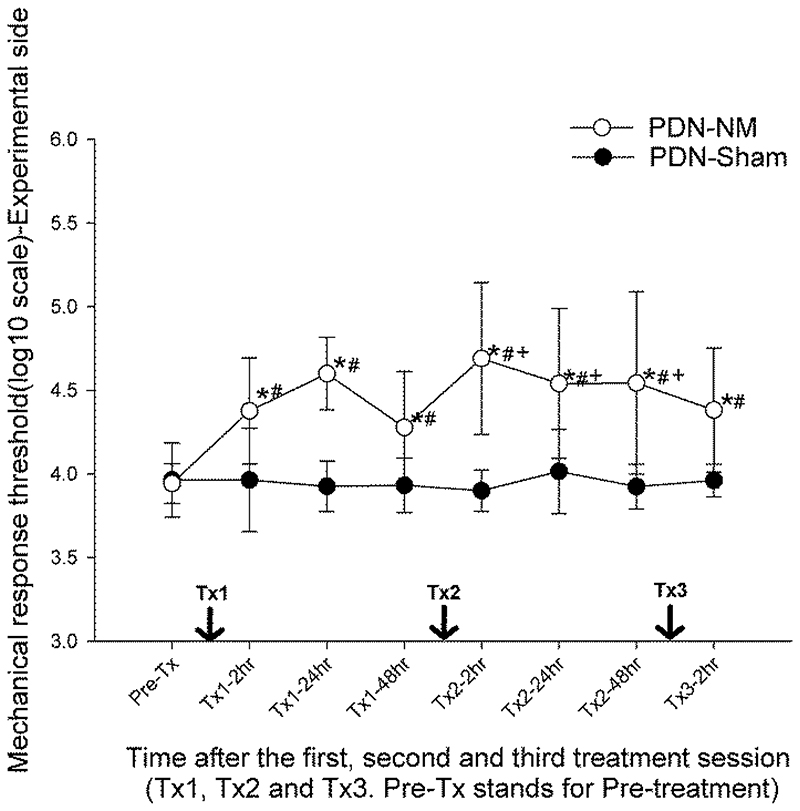

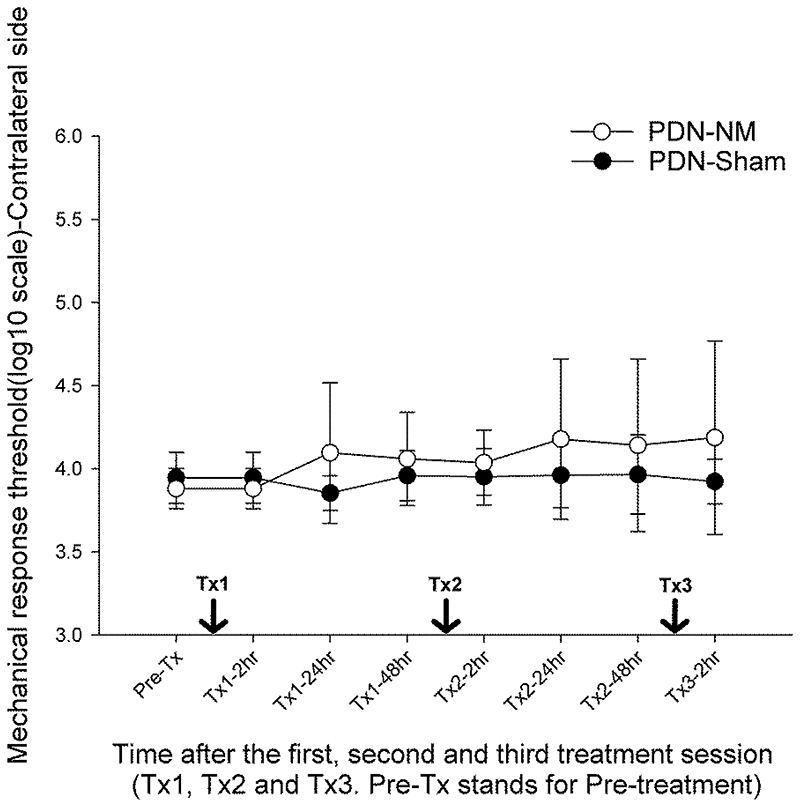

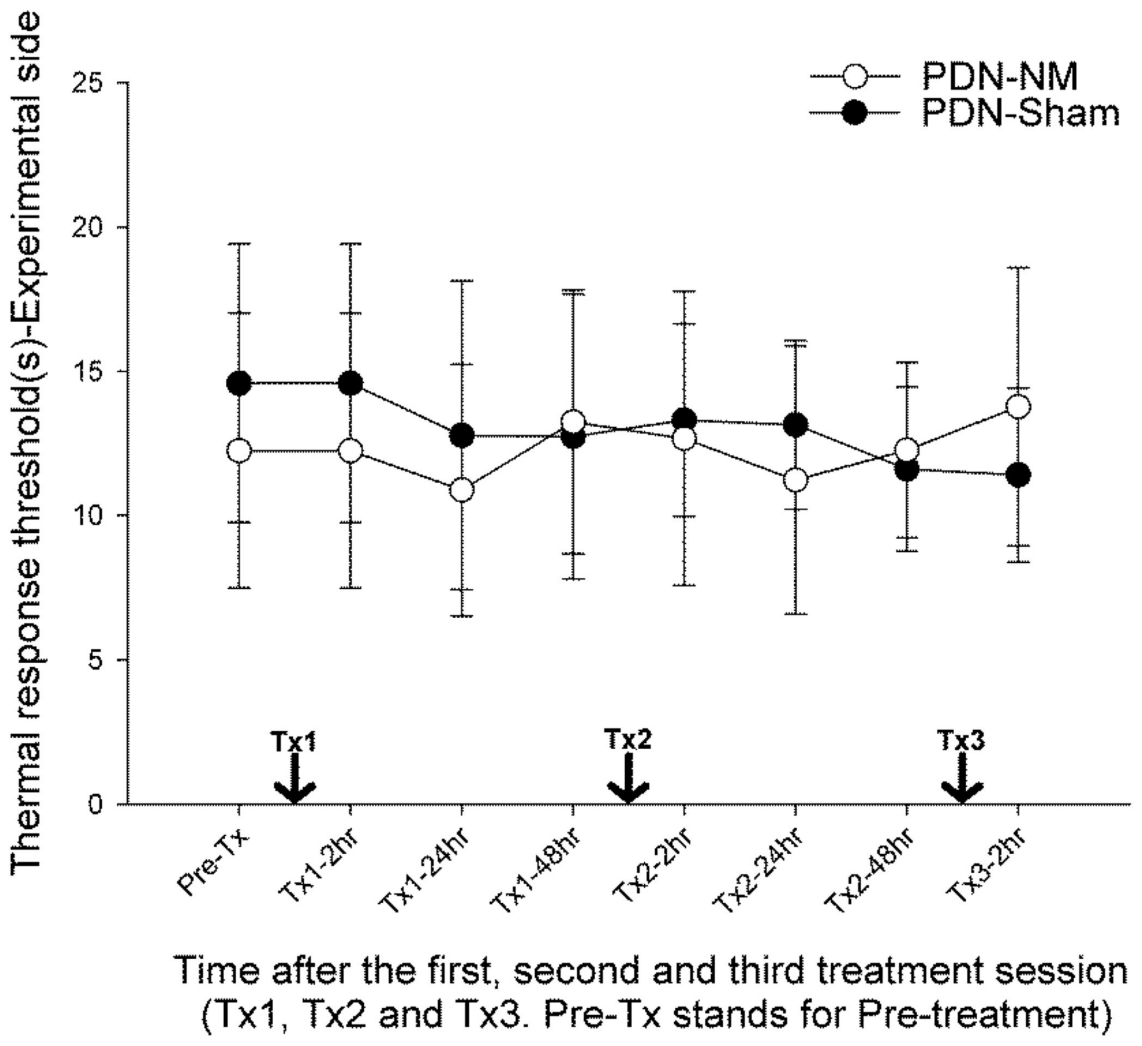

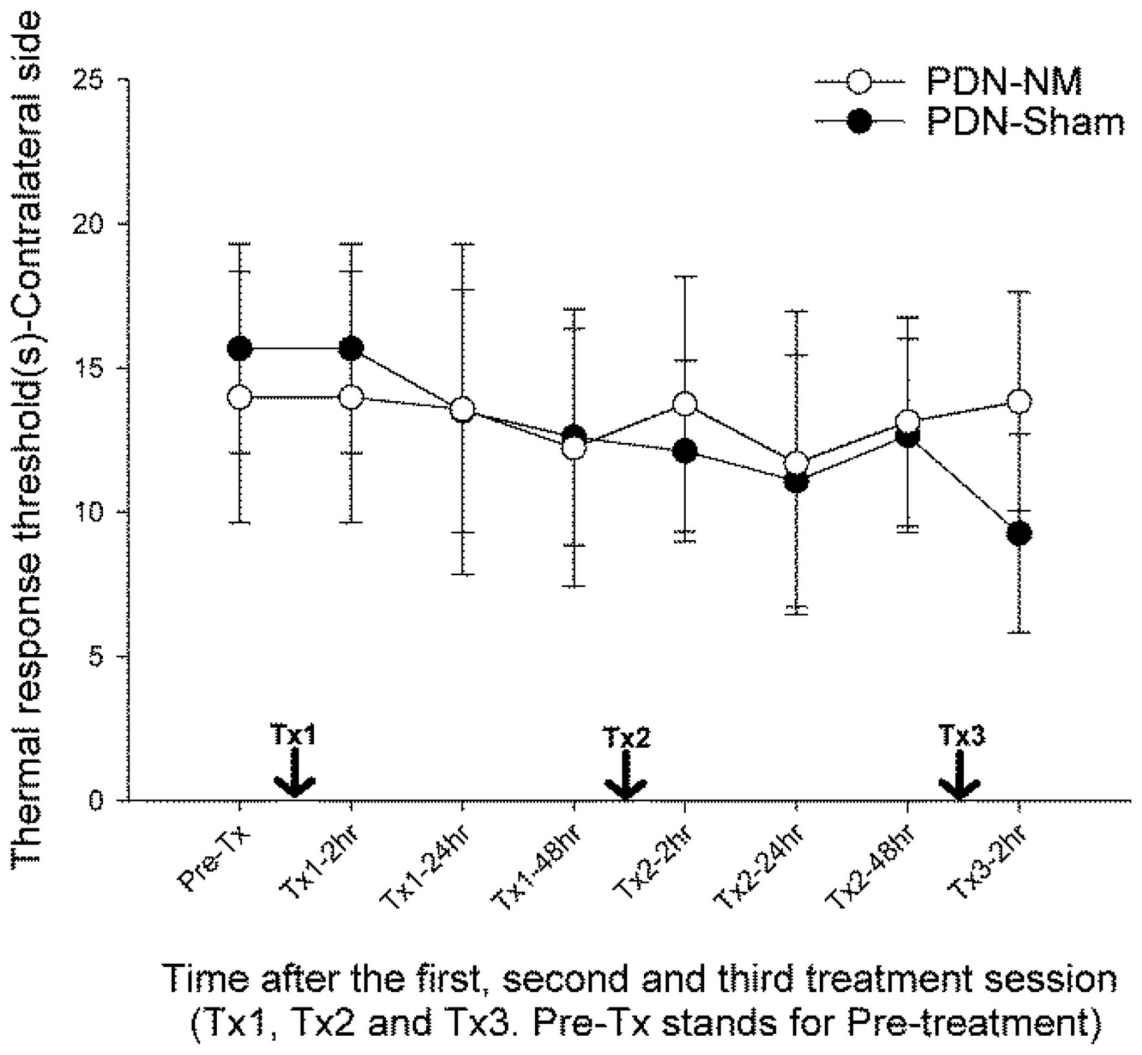
Fine-grained behavior data in relation to intervention time points. von Frey (A,B) and Plantar (C,D) test data from the experimental (A,C) and contralateral (B,D) side representing the mechanical response threshold (log(10) scale) and thermal response latency (s). The x-axis represents time after treatment sessions. Tx1, 2 and 3 represent first, second and third treatment sessions. Data are presented as mean ± standard deviations with 9 rats in PDN-Sham and 10 in PDN-NM group. The mechanical response threshold on the experimental side of the PDN-NM group increased after the first NM session and was maintained for 48 hours. The second session further increased the mechanical response threshold and was also maintained for 48 hours (A). The third NM session did not further increase but maintain the mechanical response threshold (A). On the contralateral side, no significant differences between PDN-NM and PDN-Sham group were apparent (B). No changes were observed between groups for thermal testing. *P< .05 compared to pretreatment data, +*P* < .05 compared to behavioral data from 48 hours after first treatment session, #*P* < .05 compared to PDN-Sham group at the same timepoint.

The fine-grained behavioral data of the PDN-NM and PDN-Sham groups following treatments (time-course test) was analyzed separately with 2-way (time, group) mixed ANOVA followed by post-hoc Fisher tests to evaluate the effect duration of a single NM session and the potentiation effect of multiple NM sessions. The results revealed a significant time-by-group interaction effect (*P* = .001) for the mechanical response threshold on the experimental side. Post-hoc Fisher analysis within group over time showed that the mechanical response threshold on the experimental side of the PDN-NM group was significantly increased at 2 hours after the first treatment (*P* < .001 compared to pretreatment data, Fig. 3A). The increased mechanical response threshold was maintained at 24/48 hours after the first treatment (*P* < .001/*P* = .007 compared to pretreatment data). After the second treatment session, the mechanical response threshold on the experimental side of the PDN-NM group was further increased at 2 hours after second treatment (*P* < .001 compared to 48 hours after the first treatment) and the increased threshold was maintained at 24/48 hours after the second treatment (*P* = .048/0.037 compared to 48 hours after the first treatment, Fig. 3A).

The third NM session did not further increase the mechanical response threshold. But maintained the elevated mechanical response threshold compared to pretreatment data (*P* < .001 compared to pretreatment data). In the PDN-Sham group, there were no significant changes in the mechanical response threshold after treatment (Fig. 3A, 3B).

Post-hoc Fisher test was also used to compare the mechanical response threshold between PDN-Sham and PDN-NM groups at different timepoints. The results confirmed that on the experimental side, the mechanical response threshold of the PDN-NM group was significantly higher compared to the PDN-Sham group at all post-treatment timepoints. (*P* = .011, *P* < .001 and *P* = .012; *P* < .001, *P* = .007 and *P* = .004 at 2/24/48 hours after the first/second treatment session; *P* = .004 at 2 hours after the third treatment session).

The mixed ANOVA showed no significant time-by-group interaction for mechanical response threshold on the contralateral side (*P* = .344) or thermal response threshold on experimental/contralateral sides (*P* = .107/*P* = .177) (Fig. 3B-D). These findings suggest that a single NM session can produce an ipsilateral analgesic effect on nociceptive mechanical but not thermal stimuli in rats with PDN which is maintained for 48 hours. A second treatment session further improves the effect of the first treatment, while a third treatment session maintains the treatment effect.

Data of the time-course test are summarized in Supplementary Table 2.

### NM results in higher PGP9.5+ IENFD on the experimental side

The 1-way ANOVA revealed a significant group difference for IENFD (*P* < .001). Post-hoc Fisher test showed a reduced IENFD in the PDN-Sham group on both sides compared to the Vehicle group (*P* < .001). This confirms that PDN induced significant bilateral degeneration of epidermal nerve fibers as previously reported.^27, 47^

**Figure 4:**
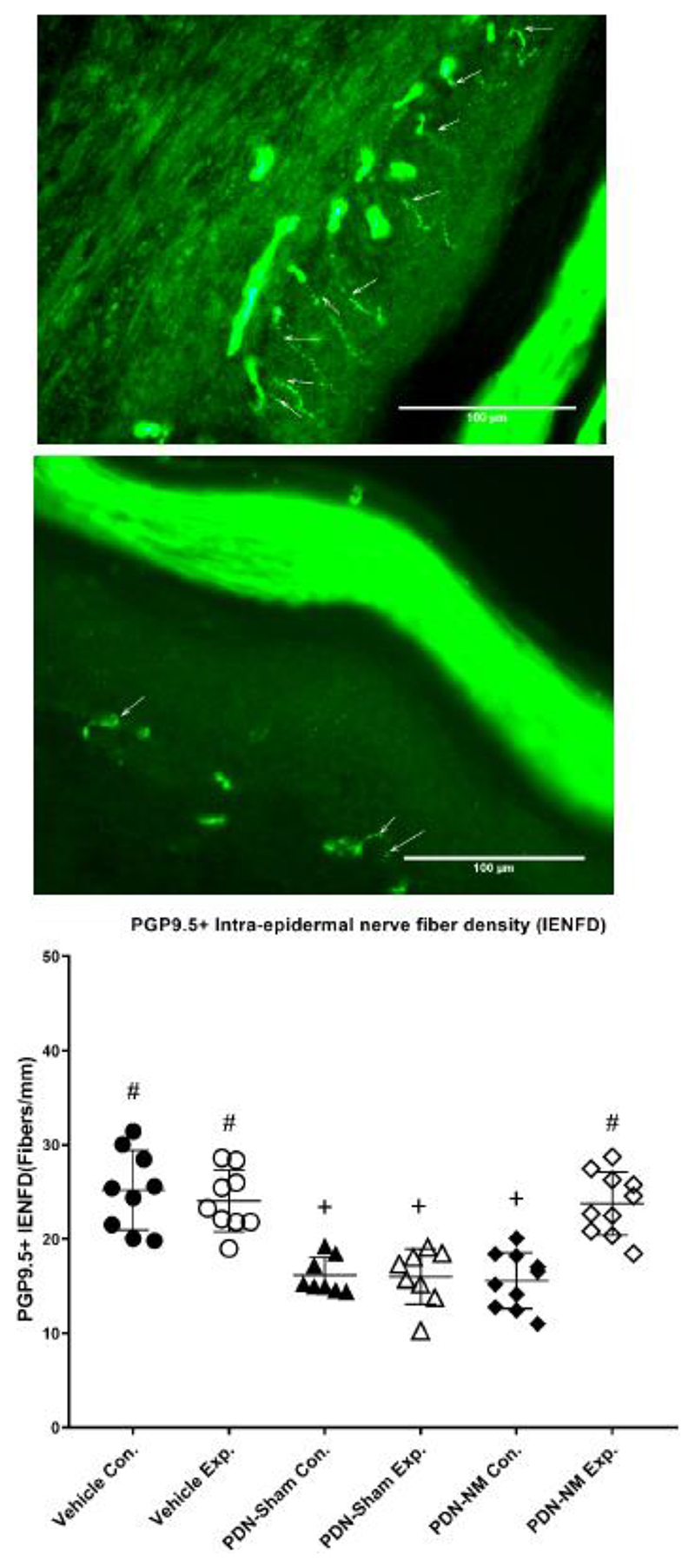
PGP9.5+ intraepidermal nerve fiber density for the glabrous paw skin. Figures (A) and (B) represent the PGP9.5 immunohistochemical staining of a representative skin section on the experimental (A) and contralateral side (B) of an animal in the PDN-NM group. Arrows indicate PGP9.5+ nerve fibers crossing the dermal/epidermal border into the epidermis. Figure C illustrate the quantification of PGP9.5+ IENFD of the experimental and contralateral paw skin in the Vehicle, PDN-Sham and PDN-NM groups. Data are presented as mean ± standard deviation for 9 rats in the Vehicle, 8 rats in the PDN-Sham and 10 rats in the PDN-NM group. Whereas IENFD was reduced in both experimental and contralateral sides of the PDN-Sham group and the contralateral side of PDN-NM group, the IENFD on the experimental side of the PDN-NM group was comparable to the Vehicle group. Con.= contralateral side, Exp.= experiment side. + *P* < .05 compared to the same side of the Vehicle group, # *P* < .05 compared to the same side of the PDN-Sham group.

The PGP9.5+ IENFD of PDN-NM group was comparable to the PDN-Sham group on the contralateral side (*P* = .704) but significantly higher compared to PDN-Sham group on the experimental side (*P* < .001). The PGP9.5+ IENFD on the experimental side of PDN-NM group was also comparable to the Vehicle group (*P* = .846) (Fig. 4C). This indicates that the NM treatment may mitigate the degeneration of IENFD in rats with PDN.

IENFD data are summarized in Supplementary Table 3.

## Discussion

Our findings demonstrate that NM reduces mechanical but not thermal hyperalgesia in rats with PDN. The beneficial effect on mechanical hyperalgesia after a single NM session was maintained for 48 hours and could be potentiated with a second treatment session while the third treatment session maintained the positive effect. Intriguingly, NM mitigated axon degeneration as apparent by the higher IENFD compared to animals receiving sham treatment. These effects were specific to the experimental but not contralateral side.

The attenuation of mechanical hyperalgesia through NM in rats with PDN replicates the findings from our previous study.^28^ However, there were no significant changes in thermal response thresholds after NM. In a previous study in rats with focal nerve injury, NM could also mitigate thermal hyperalgesia.^38^ Unlike in focal nerve injury, the thermal response threshold in rats with STZ-induced PDN remained normal throughout the study. A similar absence of thermal hyperalgesia was also reported in other studies utilizing the STZ model.^28, 48^ The lack of effect of NM on thermal response threshold in this study may therefore be attributed to the inability to change already normal thermal pain thresholds rather than the ineffectiveness of NM on thermal hyperalgesia.

Regarding the temporal pattern of behavioral responses associated with NM interventions, the result of the time-course test revealed that the effect of a single NM session could be maintained for 48 hours, and multiple treatment sessions were needed to potentiate and maintain treatment effects. To our knowledge, this study is the first to perform a fine-grained analysis on the effect duration of single NM sessions. The finding that the effect of a single NM session is maintained for 48 hours could serve as a reference for the design of future treatment programs. Previous studies that utilized NM on rats with focal nerve injury showed significant improvement in sensory and motor parameters after receiving multiple treatment sessions.^37, 38^ These studies also found a dose-dependent improvement in mechanical/thermal hypersensitivity.^37, 38^ Our results suggest that a second session potentiates the hypoalgesic effect whereas a third session maintains this effect. Although NM has been used in clinics and studied for some time, there is no standardized guideline for designing treatment programs. A recent systemic review indicated that clinical NM programs still vary significantly in technique, frequency of treatment and total length of the treatment program.^49^ By providing evidence regarding the effect duration and additive effect of multiple treatment sessions, our findings might contribute to the development of a standardized treatment program. Further experiments with longer treatment periods, different treatment frequencies and long-term follow ups will help to shed more light on optimal NM programs.

Excitingly, our results demonstrate that NM preserves IENFD in rats with PDN. This effect was specific to the experimental side. Previous studies had investigated the effects of NM on peripheral nerve degeneration in preclinical models of focal nerve injury.^32, 33^ Findings in those studies showed that animals with focal nerve injury demonstrated improved myelin integrity, increased numbers of regenerated nerve fibers and increased numbers of regenerating sprouts after NM.^32, 33^ Those findings suggest that that NM intervention may facilitate regeneration of injured nerve fibers. Here, the time frame between the start of NM interventions and the collection of skin samples was relatively short (5 days). In this time, an increase in 7.5 fibers/mm was apparent compared to the sham intervention. It is unlikely that the observed increase in IENFD was solely the result of nerve regeneration. In humans, the regenerative capacity of intraepidermal nerve fibers following experimental denervation is 0.17 fibers/mm/day.^50^ The rate was reduced in patients with diabetic polyneuropathy (0.074 fibers/mm/day).^50^ Thus, the preserved IENFD following NM is unlikely due to an isolated regenerative effect of NM, but may represent mitigation of degeneration. This hypothesis is further supported by the concurrent hypoalgesic effect. Regeneration of unmyelinated fibers during the degeneration process has been associated with hypersensitivity in PDN rather than hypoalgesia.^24, 27^ On the other hand, progressive loss of IENF may be associated with the development of neuropathic pain in early diabetic neuropathy.^22^ As such, the mitigation of axonal degeneration and hypoalgesia observed here may be driven by the prevention of secondary mechanisms such as hyper-excitability of surviving sensory neurons or spinal disinhibition.^10, 51^

Our preclinical findings may have important implications for clinical practice. It remains challenging to alter the progression of a developed diabetic neuropathy, especially in patients with type 2 diabetes.^20, 52, 53^ Our results suggest that NM may play a role in the prevention or slowing of PDN if implemented early.

## Limitations

Although this preclinical study provided some valuable findings, it remains to be examined whether similar effects of NM can be reproduced in patients with diabetes. The STZ model is 1 of the most frequently used preclinical models for PDN, and is modelling the less common type 1 diabetes. While hyperalgesia and nerve degeneration are dominant mechanisms in both diabetes types,^54, 55^ it remains to be explored whether the effect of NM shown here can be replicated in the context of type 2 diabetes. We chose a relatively short timeframe and follow up in an experimental model of early PDN to study the effects of NM. Future studies will have to explore whether NM has long-term benefit that can be maintained even after treatment has ceased in early PDN and whether similar effects can be elicited in chronic stages.

There are also 2 methodological considerations. First, we included only male animals. Although this minimizes the potential influence of the estrous cycle on pain sensitivity^39^, it limits the generalizability of our findings. Sex differences are apparent in preclinical models of neuropathic pain.^56^ Also, women with diabetes are at higher risk of developing PDN and report higher pain severity.^57–59^ Second, we did not use the commonly accepted up-down method for von Frey filament testing.^60^ Instead, we used the simplified up-down method (SUDO)^41^ which requires fewer stimulations, reducing the total test time. While the validity of this method needs to be examined further, we have previously used this method in our studies with similar results.^28, 29, 42^

## Conclusions

Our study revealed that a single NM session can mitigate mechanical allodynia in rats with PDN. Multiple treatment sessions were required to potentiate and maintain this treatment effect. In addition, histological analysis suggests that NM can mitigate the degeneration of sensory axon terminals. Further studies are needed to develop optimal NM paradigms, and to translate these findings to patients with diabetic neuropathy.

## Supplementary Material

1-3

## Figures and Tables

**Table 1:** List of Antibodies Used for Immunohistochemistry Analysis

Antibody	Concentration	Source
Mouse anti-human Protein Gene Product 9.5 (PGP9.5) clone 31A3	1:200	Bio-Rad Laboratories Inc (Hercules, California, USA) Catalog no. 7863-1004
Horse anti-mouse Biotinlyated antibody	1:100	Vector Laboratories Inc (California, USA) Catalog no. BA2001
Streptavidin Alexa 488 Secondary antibody	1:500	Invitrogen/Thermo Fisher Scientific Corp (Carlsbad, California, USA) Catalog no. S11223
