# Supplementary material for: Effects of Neural Mobilization on Sensory Dysfunction and Peripheral Nerve Degeneration in Rats With Painful Diabetic Neuropathy
Source: Phys Ther. Author manuscript; Available in PMC 2022 Oct 6. (PMC7613682; doi:10.1093/ptj/pzac104)
Supplement: 1-3 [file EMS152408-supplement-1_3.docx]

Supplementary table 1. Summary of daily behavioral data. Mechanical (in log(10) scale) and thermal response thresholds (in seconds) are presented as mean ± standard deviation. Baseline: before STZ/saline injection, D3-14: days 3-14 after STZ/saline injection, PDN: painful diabetic neuropathy; NM: neural mobilization

Daily behavioral data

| Vehicle group | Baseline | D3 | D4 | D5 | D6 | D7 | D10 | D11 | D12 | D13 | D14 |
| --- | --- | --- | --- | --- | --- | --- | --- | --- | --- | --- | --- |
| Mechanical response threshold  Experimental side | 5.37±0.05 | 5.35±0.11 | 5.31±0.16 | 5.34±0.16 | 5.33±0.11 | 5.30±0.25 | 5.31±0.15 | 5.36±0.12 | 5.32±0.16 | 5.34±0.08 | 5.36±0.08 |
| Mechanical response threshold  Contralateral side | 5.36±0.08 | 5.38±0.06 | 5.36±0.11 | 5.40±0.05 | 5.36±0.07 | 5.37±0.08 | 5.36±0.10 | 5.35±0.14 | 5.38±0.07 | 5.38±0.07 | 5.33±0.12 |
| Thermal response threshold-  Experimental side | 15.64±3.55 | 14.63±3.81 | 14.06±4.18 | 15.53±3.37 | 12.32±4.51 | 11.23±5.70 | 10.48±4.70 | 10.10±3.80 | 10.02±5.86 | 12.45±3.51 | 12.11±3.08 |
| Thermal response threshold  Contralateral side | 15.75±2.97 | 13.96±3.34 | 13.94±3.65 | 14.26±3.09 | 13.41±4.96 | 10.21±4.24 | 11.12±4.65 | 10.84±4.56 | 9.07±4.19 | 13.86±5.07 | 14.58±4.13 |

| PDN-Sham group | Baseline | D3 | D4 | D5 | D6 | D7 | D10 | D11 | D12 | D13 | D14 |
| --- | --- | --- | --- | --- | --- | --- | --- | --- | --- | --- | --- |
| Mechanical response threshold  Experimental side | 5.37±0.07 | 3.96±0.20 | 4.02±0.21 | 4.17±0.56 | 3.96±0.18 | 4.18±0.33 | 3.96±0.31 | 3.93±0.15 | 3.90±0.12 | 4.01±0.25 | 3.96±0.10 |
| Mechanical response threshold  Contralateral side | 5.38±0.07 | 4.01±0.29 | 3.97±0.23 | 4.18±0.49 | 4.14±0.30 | 4.23±0.38 | 3.85±0.10 | 3.96±0.15 | 3.96±0.19 | 3.96±0.24 | 3.95±0.12 |
| Thermal response threshold-  Experimental side | 15.04±4.32 | 14.21±2.91 | 12.43±4.41 | 14.01±4.13 | 12.29±2.89 | 13.27±3.37 | 12.78±5.34 | 12.74±4.93 | 13.14±2.92 | 11.61±2.85 | 14.60±2.84 |
| Thermal response threshold  Contralateral side | 15.20±3.43 | 13.17±3.95 | 12.65±4.94 | 12.08±4.46 | 12.71±4.53 | 14.35±3.89 | 13.51±4.21 | 12.61±3.75 | 11.09±4.36 | 12.66±3.36 | 12.06±4.31 |

| PDN-NM group | Baseline | D3 | D4 | D5 | D6 | D7 | D10 | D11 | D12 | D13 | D14 |
| --- | --- | --- | --- | --- | --- | --- | --- | --- | --- | --- | --- |
| Mechanical response threshold  Experimental side | 5.30±0.10 | 4.18±0.44 | 3.99±0.18 | 4.18±0.23 | 4.06±0.15 | 3.99±0.19 | 4.38±0.32 | 4.60±0.22 | 4.69±0.45 | 4.54±0.45 | 4.38±0.37 |
| Mechanical response threshold  Contralateral side | 5.28±0.10 | 4.17±0.49 | 4.04±0.23 | 4.14±0.20 | 4.04±0.19 | 3.96±0.11 | 4.09±0.42 | 4.06±0.28 | 4.18±0.48 | 4.14±0.52 | 4.00±0.24 |
| Thermal response threshold-  Experimental side | 15.09±3.29 | 14.1 ±3.60 | 13.70±5.49 | 13.52±3.72 | 11.96±2.52 | 13.62±4.61 | 10.88±4.36 | 13.24±4.57 | 11.23±4.65 | 12.26±3.04 | 10.61±5.00 |
| Thermal response threshold  Contralateral side | 13.67±3.78 | 15.06±4.39 | 12.72±5.05 | 13.35±3.88 | 12.17±4.42 | 14.91±4.41 | 13.57±5.73 | 12.23±4.80 | 11.70±5.25 | 13.13±3.62 | 13.25±3.39 |

Supplementary table 2. Summary of behavioral data in the time course experiment. Mechanical (in log(10) scale) and thermal response thresholds (in seconds) are presented as mean ± standard deviation. Pre-Tx: time point right before treatment (NM or Sham) start, tx1-tx3: First to third treatment, 2hr-48hr: 2-48 hours after the respective treatment session, PDN: painful diabetic neuropathy; NM: neural mobilization

| PDN-NM | Pre-Tx | tx1-2hr | tx1-24hr | tx1-48hr | tx2-2hr | tx2-24hr | tx2-48hr | tx3-2hr |
| --- | --- | --- | --- | --- | --- | --- | --- | --- |
| Mechanical response threshold  Experimental side | 3.94±0.12 | 4.38±0.32 | 4.60±0.22 | 4.28±0.33 | 4.69±0.45 | 4.54±0.45 | 4.54±0.55 | 4.38±0.37 |
| Mechanical response threshold  Contralateral side | 3.88±0.12 | 4.09±0.42 | 4.06±0.28 | 4.04±0.20 | 4.18±0.48 | 4.14±0.52 | 4.19±0.58 | 4.00±0.24 |
| Thermal response threshold-  Experimental side | 12.25±4.77 | 10.88±4.36 | 13.24±4.57 | 12.67±5.10 | 11.23±4.65 | 12.26±3.04 | 13.77±4.83 | 10.61±5.00 |
| Thermal response threshold  Contralateral side | 13.99±4.36 | 13.57±5.73 | 12.23±4.80 | 13.75±4.42 | 11.70±5.25 | 13.13±3.62 | 13.84±3.79 | 13.25±3.39 |

| PDN-Sham | Pre-Tx | tx1-2hr | tx1-24hr | tx1-48hr | tx2-2hr | tx2-24hr | tx2-48hr | tx3-2hr |
| --- | --- | --- | --- | --- | --- | --- | --- | --- |
| Mechanical response threshold  Experimental side | 3.96±0.22 | 3.96±0.31 | 3.93±0.15 | 3.93±0.16 | 3.90±0.12 | 4.01±0.25 | 3.92±0.13 | 3.96±0.10 |
| Mechanical response threshold  Contralateral side | 3.95±0.15 | 3.85±0.10 | 3.96±0.15 | 3.95±0.17 | 3.96±0.19 | 3.96±0.24 | 3.92±0.13 | 3.95±0.12 |
| Thermal response threshold-  Experimental side | 14.58±4.83 | 12.78±5.34 | 12.74±4.93 | 13.31±3.34 | 13.14±2.92 | 11.61±2.85 | 11.40±3.01 | 14.60±2.84 |
| Thermal response threshold  Contralateral side | 15.68±3.62 | 13.51±4.21 | 12.61±3.75 | 12.12±3.14 | 11.09±4.36 | 12.66±3.36 | 9.26±3.44 | 12.06±4.31 |

Supplementary table 3. Summary of intraepidermal nerve fiber density (IENFD) data presented as mean ± standard deviation. PDN: painful diabetic neuropathy; NM: neural mobilization

|  | Vehicle | PDN-Sham | PDN-NM |
| --- | --- | --- | --- |
| IENFD-  Experimental side | 24.04±3.26 | 15.99±2.94 | 23.75±3.34 |
| IENFD-  Contralateral side | 25.17±4.22 | 16.15±1.89 | 15.57±2.97 |
